# Targeted Methylation of the Epithelial Cell Adhesion Molecule (EpCAM) Promoter to Silence Its Expression in Ovarian Cancer Cells

**DOI:** 10.1371/journal.pone.0087703

**Published:** 2014-01-29

**Authors:** Suneetha Nunna, Richard Reinhardt, Sergey Ragozin, Albert Jeltsch

**Affiliations:** 1 Institute of Biochemistry, Stuttgart University, Stuttgart, Germany; 2 Max-Planck-Genomzentrum Köln, Köln, Germany; CEA - Institut de Genomique, France

## Abstract

The Epithelial Cell Adhesion Molecule (EpCAM) is overexpressed in many cancers including ovarian cancer and EpCAM overexpression correlates with decreased survival of patients. It was the aim of this study to achieve a targeted methylation of the EpCAM promoter and silence EpCAM gene expression using an engineered zinc finger protein that specifically binds the EpCAM promoter fused to the catalytic domain of the Dnmt3a DNA methyltransferase. We show that transient transfection of this construct increased the methylation of the EpCAM promoter in SKOV3 cells from 4–8% in untreated cells to 30%. Up to 48% methylation was observed in stable cell lines which express the chimeric methyltransferase. Control experiments confirmed that the methylation was dependent on the fusion of the Zinc finger and the methyltransferase domains and specific for the target region. The stable cell lines with methylated EpCAM promoter showed a 60–80% reduction of EpCAM expression as determined at mRNA and protein level and exhibited a significantly reduced cell proliferation. Our data indicate that targeted methylation of the EpCAM promoter could be an approach in the therapy of EpCAM overexpressing cancers.

## Introduction

Cancer occurring in the peritoneal cavity of the ovaries is the seventh most common cancer in women and second leading cause of death worldwide among gynecological cancers [1]–[3]. In most women, ovarian cancer is difficult to treat with a five year survival rate of around 20% in cancers diagnosed in advanced stage [4]–[6]. Platinum-based analogues such as Cisplatin or Carboplatin are the major standard chemotherapy agents to treat ovarian cancer in initial stages [7]. However, their use is hindered by the acquired or intrinsic resistance of the cancer cells to the drug [8]. In spite of an increased understanding in the etiology of ovarian cancer there has been little change in the survival of patients over the past 30 years, because in the early stages ovarian cancer is asymptomatic and there are no efficient tumor specific and sensitive markers to monitor epithelial ovarian cancer [9]. Thus, there is an immediate need for new strategies for the treatment of ovarian cancer.

Ovarian cancer cells exhibit over expression of the Epithelial Cell Adhesion Molecule (EpCAM) when compared with normal ovarian cells [10]–[14]. EpCAM (NCBI Reference Sequence NM_002354.2; also called GA733, KSA, 17-1A antigen, or CD326) is a 40 kDa epithelial cell surface glycoprotein that mediates Ca^2+^ independent homophilic cell-cell adhesion [10], [15], [16]. The epithelium of healthy individuals expresses EpCAM, with the exception of squamous epithelium and of specific epithelial cells of adult hepatocytes and keratinocytes [17]. EpCAM is over-expressed to varying degrees in numerous human carcinomas [18], [19], cancer-initiating cells, and in progenitor and normal stem cells [20]. It has recently been shown that EpCAM upregulates *c-myc, cyclin A* and *E* and it influences the cell cycle and enhances cell proliferation [21]. In addition, it is involved in the nuclear Wnt-signaling pathway that also promotes cell proliferation and tumorigenesis [20].

Though the exact role of EpCAM is elusive in ovarian cancer progression, the EpCAM over expression significantly correlates with decreased survival rate in patients at stage III/IV of the disease and over expression of EpCAM in breast and gallbladder cancer has a strong correlation with poor prognosis [22]–[24]. Anti-EpCAM antibodies were used to identify circulating tumor cells in the blood of cancer patients, and to provide prognostic information that allows treatment of patients [25]. In addition, the direct association of EpCAM with the progression of ovarian cancer suggested that it may serve as potential therapeutic target for the treatment of ovarian cancer and different approaches have been established to target EpCAM [26], [27]. EpCAM antibodies such as MT201 efficiently eliminate cancer cells from ovarian cancer patients [28]. For example, Catumaxomab has been approved for the treatment of malignant ascites and it has been used for epithelial ovarian and non-ovarian cancers [29]–[31]. Although, anti-EpCAM monoclonal antibodies provide protection against cancer [32], [33], the antibody dependent cytotoxicity relies on the CH2 domain of the antibody that varies significantly from batch to batch during antibody production [34]. In addition, anti-EpCAM antibodies failed to provide any clinical protection against colorectal and prostate cancer due to the large size of the antibody which confines distribution and delivery [34]–[36]. Hence, better and more general strategies for the targeted repression of EpCAM are required.

As an alternative approach, the oncogenic function of EpCAM was inhibited by reducing the expression of its gene. One method to achieve this was the application of antisense RNA which has led to a strong decrease in cell proliferation and metabolism in human carcinoma cells [21]. In a similar approach, siRNA mediated silencing of EpCAM expression strongly reduced the cell migration and invasive potential of breast cancer cells [23]. EpCAM expression was also silenced by the expression of a Zinc-finger protein which binds to the EpCAM promoter [37] and a fusion of an EpCAM targeting Zinc finger domain with a repressor domain [38]. However, all these approaches did not lead to a persistent down regulation which stimulated attempts to connect gene repression with epigenetic marks, like DNA methylation. DNA methylation plays an important role in epigenetic regulation of gene expression [39]–[42]. In mammals, DNA methylation occurs at C5 position of the cytosine mainly in the context of CpG dinucleotides. The CpG rich regions are termed CpG islands and often occur in gene promoter regions [43]. The EpCAM promoter contains a CpG island, the methylation of which inversely correlates with EpCAM expression, tumor invasion and progression in various cancer types [13], [44]. In support of this concept, it could be shown that DNA methylation introduced at the promoter in an undirected fashion led to down regulation of the EpCAM gene [45].

**Figure 1:**
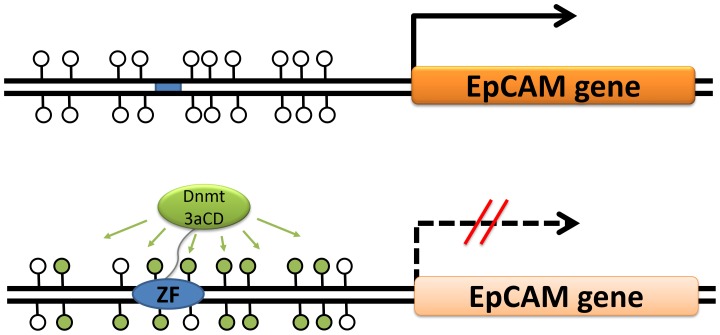
Principle of targeted DNA methylation and gene silencing using Zinc fingers (ZF) fused to the catalytic domain of the DNA methyltransferase Dnmt3a (Dnmt3aCD). The blue bar represents the ZF binding site, unfilled lollipops represent unmethylated CpGs and filled lollipops represent methylated CpGs.

Clearly, to reduce side effects of an epigenetic gene silencing treatment, a targeted delivery of silencing epigenetic marks like DNA methylation is needed. This requires the coupling of a catalytic moiety which delivers the silencing signal with a targeting part, which ensures the specific binding of the catalytic entity to a defined genomic target [46]–[48] (Fig. 1). Different molecular entities are in use for the sequence specific targeting function, including fusion of the DNA methyltransferase part to a triplex forming oligonucleotide [49], fusion to engineered Zinc finger proteins (see below), TAL effectors [50], [51] and in future potentially engineered CRISPR/CAS systems [52]. Since Zinc fingers were the first protein modules for which design of sequence specific DNA binding became possible [53]–[58], initial targeted methylation studies relied on artificial zinc-finger proteins as targeting device [59]. Following this approach, the repression of viral genes [60], down-regulation of Maspin and SOX2 [61] and the VEGFA gene suppression [62] has been achieved in human cell lines. In this work, we fused a Zinc finger protein that targets the EpCAM promoter [37] to the DNA methyltransferase 3a (Dnmt3a) catalytic domain in order to methylate the EpCAM promoter, which subsequently should lead to EpCAM silencing. We used SKOV3 cells which are derived from human female adenocarcinoma cells and have been used as a model for ovarian cancer [45]. We show that the targeted methylation of the promoter leads to reduction of the EpCAM gene expression, further supporting the notion that targeted DNA methylation is a general approach to gene silencing. Furthermore, we show that silencing of EpCAM leads to a reduction in proliferation of SKOV3 cells.

## Materials and Methods

### Cell culture, transfection and enrichment of transfection

SKOV3 human ovarian cancer cells were obtained from ATCC (American type culture collection). The cells were cultured in Dulbecco's modified Eagle's medium (PAA) supplemented with 10% fetal bovine serum and 2 mM L-glutamine (PAA). For transient transfection, 3.5×10^5^ cells were seeded in T25 flasks and the cells were transfected after one day with 12 µl of FuGENE HD transfection reagent (Promega) and 6 µg of three plasmid DNAs which express one the different Zinc finger fusion constructs used in this work, the LNGFR marker, and GFP in a ratio of 10∶2.5∶1 as previously described [62]. The transfected cells were enriched using MACSelect™ LNGFR system (Miltenyi Biotec) according to the manufacturer's instructions. Briefly, when cells are co-transfected with the LNGFR molecule, it is expressed on the cell surface. After four days, cells were incubated with magnetic beads conjugated with anti-LNGFR antibody for 15 minutes at 4°C. Transfected cells were passed through a MACS separation column placed in a MACS separator and washed twice with PBE buffer (Phosphate buffer saline with 0.5% bovine serum albumin and 5 mM EDTA) to wash away the untransfected cells. After washing, only transfected cells bound by magnetic beads are retained in the column and the enriched cells were eluted in PBE and used for further methylation analysis.

For stable cell line generation, 2×10^5^ cells were seeded per well in 6 well plates, and the cells were transfected with 6 µl of FuGENE HD transfection reagent and 2 µg of EpCAM ZF-Dnmt3aCD plasmid DNA. After 48 h, the cells were grown in medium containing 800 ng/ml G418 for six weeks. Monoclonal colonies were obtained and further expanded in the presence of G418 containing medium and two of the stable cell lines were used for further experiments.

### Methylation analysis of the EpCAM promoter and non-target gene methylation

Genomic DNA was isolated using QIAamp® DNA mini kit (QIAGEN), and bisulfite conversion was carried out as described previously [63]. Briefly 400 ng of genomic DNA was digested with SphI restriction enzyme overnight at 37°C. Bisulfite conversion was carried out in the presence of NaOH and sodium bisulfite using the following conditions: 15 min incubation at 99°C, 30 min at 50°C, 5 min at 99°C, 1.5 h at 50°C, 5 min at 99°C and 1.5 h at 50°C. During the incubation with bisulfite, unmethylated cytosine is converted to uracil but methylated cytosines remain unchanged. Afterwards, the DNA was concentrated and purified using Amicon ultra centrifugal filters (Millipore) and PCR amplified using bisulfite specific primers. The uracil is amplified as Thymine in this reaction. The PCR product was subcloned using StrataClone PCR cloning kit and individual clones were sequenced. To analyze its methylation status, 220 bp of the EpCAM promoter containing 16 CpGs was amplified using specific primers (For: 5′-CTT TTT AAG GTT TTA GAG TAG-3′ and Rev: 5′-AAA AAA TAA ATA AAC TCC CCT CCC-3′). For non-target gene methylation, four amplicons from genes (KIAA0179, DSCR3, Sumo3 and WRB, respectively) were analyzed; the sequences of all amplicons and a list of primers used are given in Supplementary Information S1. Data were analyzed using the BISMA software [64].

### RT qPCR analysis

Total RNA was isolated from stable cell lines and control SKOV3 cells using Purelink™ RNA mini kit (Ambion). A total of 1.0 µg RNA was converted into complementary DNA using M-MuLV Reverse Transcriptase (NEB) with oligo dT primers. Q-RT PCR was carried out using CFXConnect™ Real-Time system (Bio-Rad). EpCAM transcript expression levels were quantified using SsoFast™ EvaGreen® supermix (Bio-Rad) and EpCAM specific primers (for: 5′-GAACAATGATGGGCTTTATG-3′, rev: 5′-TGAGAATTCAGGTGCTTTTT-3′) using beta actin as internal control (for: 5′-TCACCAACTGGGACGACATG-3′, rev: 5′-ACCGGAGTCCATCACGATG-3′). The relative amount of transcript was measured by threshold cycle amplification (C_T_) which is inversely correlated with the amount of RNA present. The fold change in EpCAM RNA was calculated using the ΔΔCt method. Ct values are determined as mean of triplicates and the experiment was carried in two technical repeats.

### Western blotting

Cell lysates were prepared from stable cell lines and control SKOV3 cells using RIPA buffer (50 mM Tris, 150 mM NaCl, 1%NP40 and 1 mM PMSF), and the total protein content was quantified by BCA™ Protein assay kit (Thermo Scientific). Four µg of total protein was resolved on 10% SDS-PAGE gels and transferred to a nitrocellulose membrane. The membrane was blocked overnight by incubation with 4% bovine serum albumin in Phosphate buffer saline, and probed with rabbit anti-human EpCAM IgG in 1∶4000 dilution (Abcam Ab71916), followed by horseradish peroxidase conjugated goat anti-rabbit IgG in 1∶4000 dilution (GE Healthcare). The blots were developed using enhanced chemiluminescence western blotting solution (Thermo Scientific), and images were captured on X-ray films, scanned, and quantified using Image J software.

### Cell Proliferation assay (CCK8 assay)

Cell proliferation assays were performed using Cell counting kit-8 (CCK8) assay (Dojindo) according to the manufacturer's instructions. Briefly, SKOV3 cells or stable cell lines CD1 and CD2 were seeded in a density of 1.5×10^4^ cells in 200 µl medium in a 96 well cell culture plate and grown for three days in DMEM with 10% FCS at 37°C with 5% CO_2_. After three days, the cells were treated with 10 µl of the CCK8 reagent per well and incubated for 4 h in the CO_2_ incubator. Then, 100 µl supernatant from each well were transferred into fresh 96 well plates and the absorbance was measured at 450 nm using spectrophotometer. The CCK8 reagent contains WST-8 [2-(2-methoxy-4-nitrophenyl)-3-(4-nitrophenyl)-5-(2,4-disulfophenyl)-2H-tetrazolium, monosodium salt] dissolved in water and cell culture medium, which can enter the cell where it is reduced by dehydrogenases to give an orange colored formazan product. The amount of formazan formed is directly proportional to the number of living cells.

### Cell counting assay

SKOV3 cells or stable cell lines CD1 and CD2 were seeded in a six well cell culture plate in a density of 2×10^5^cells per well and cultured for four days in DMEM with 10% FCS at 37°C with 5% CO_2_. After four days, the cells were washed twice with Phosphate buffer saline, trypsinized and harvested separately and a single cell suspension was prepared. Cells were diluted in Trypan blue stain 0.4% (Gibco) in a ratio of 1∶1 and the total number of viable cells in each well was counted using a Neubauer counting chamber or haemocytometer. Live cells can be differentiated from dead cells, because dead cells take up the dye and stain dark blue whereas the membranes of living cells are intact and prevent the uptake of the dye.

## Results

### Targeted DNA methylation of EpCAM promoter in ovarian cancer SKOV3 cells

**Figure 2:**
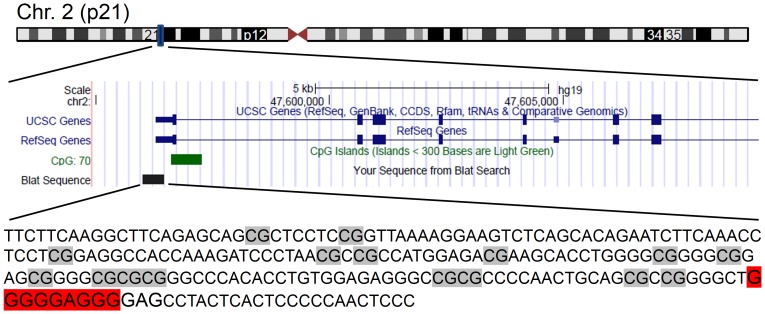
Genome context of the EpCAM gene (indicated by a blue bar) on chromosome 2 p21. The gene is shown in blue, its CpG island in green and the amplicon in black. The amplicon sequence is given below, the ZF binding site sequence is shaded in red. This picture was generated using University of California Santa Cruz genome browser (http://genome.ucsc.edu/) [66].

**Figure 3:**
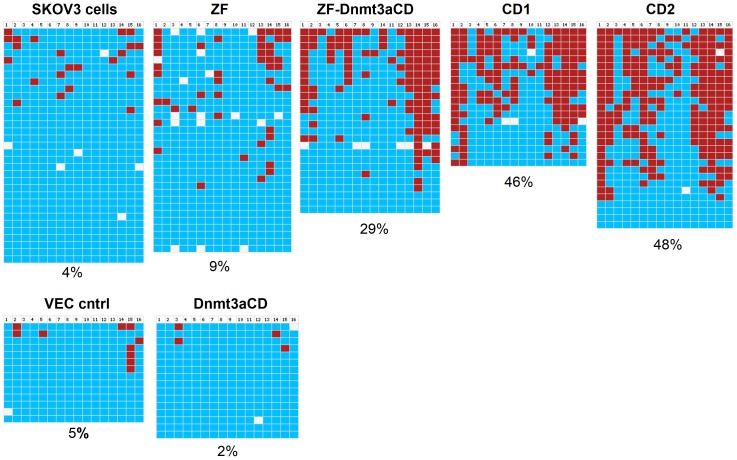
Examples of the results of the DNA methylation analysis of the EpCAM gene promoter in SKOV3 cells. The following abbreviations were used: SKOV3 cells, untreated cells; ZF, SKOV3 cells transfected with Zinc finger construct, ZF-Dnmt3aCD, cells transfected with the Zinc finger Dnmt3a catalytic domain construct; VEC cntrl, cells transfected with empty vector; Dnmt3aCD, cells transfected with a Dnmt3aCD construct without Zinc finger; CD1, stable cell line expressing ZF-Dnmt3aCD 1; CD2, stable cell line expressing ZF-Dnmt3aCD 2. The horizontal rows indicate the CpGs in the amplicon analyzed and the vertical rows represent individual clones that were sequenced. The blue and red colors represent unmethylated CpG and methylated CpG, respectively, for white colored sites, the methylation state is unknown due to technical reasons.

It was the aim of this study to achieve a targeted methylation of the EpCAM promoter and silence EpCAM gene expression. We have used an engineered zinc finger protein that specifically binds the EpCAM promoter [37] and fused it with the catalytic domain of the Dnmt3a DNA methyltransferase (Dnmt3aCD), which previously has been successfully used to introduce targeted DNA methylation [60], [62]. For the target specific methylation, SKOV3 cancer cells, which have an unmethylated EpCAM promoter and active EpCAM expression [11], were transiently transfected with the chimeric Zinc finger - Dnmt3a catalytic domain (ZF-Dnmt3aCD) constructs. After four days the transfected cells were enriched by MACS selection and the methylation status of the EpCAM promoter (Fig. 2) was analyzed by bisulfite conversion and sequencing of individual clones. In two independent experiments, untransfected SKOV3 cells showed a basal level of 4–8%DNA methylation. However, the methylation increased to 29% (±4%) in ZF-Dnmt3aCD transfected cells in two independent experiments (Fig. 3). Importantly, our data show that methylation levels of >80% could be achieved at some specific CpG sites of the target region, like the sites 14–16. The EpCAM promoter showed basal level of methylation in SKOV3 cells that were transfected with control vectors, either vector control alone, zinc finger without Dnmt3aCD or Dnmt3aCD without zinc finger, respectively (Fig. 3). The finding that the expression of untargeted Dnmt3a catalytic domain did not lead to a large increase in DNA methylation at the EpCAM promoter is in agreement with previous observations at another target locus [62].

Since we conducted the methylation experiments in transiently transfected cells after MACS selection, a background of untransfected cells was still present, which we estimate to be in the range of 20% based on microscopic observation. To obtain a homogenous cell sample, in which all cells were treated with the chimeric methyltransferase, we generated two independent stable cell lines (called CD1 and CD2), which express the Zinc finger Dnmt3aCD chimeric methyltransferase. Genomic DNA was isolated from both cell lines and the methylation status of the EpCAM promoter was analyzed as described above. Our results show that the methylation levels of EpCAM promoter were increased to 46% in the stable cell line CD1 and 48% in CD2 (Fig. 3).

### Off-target gene methylation

**Figure 4:**
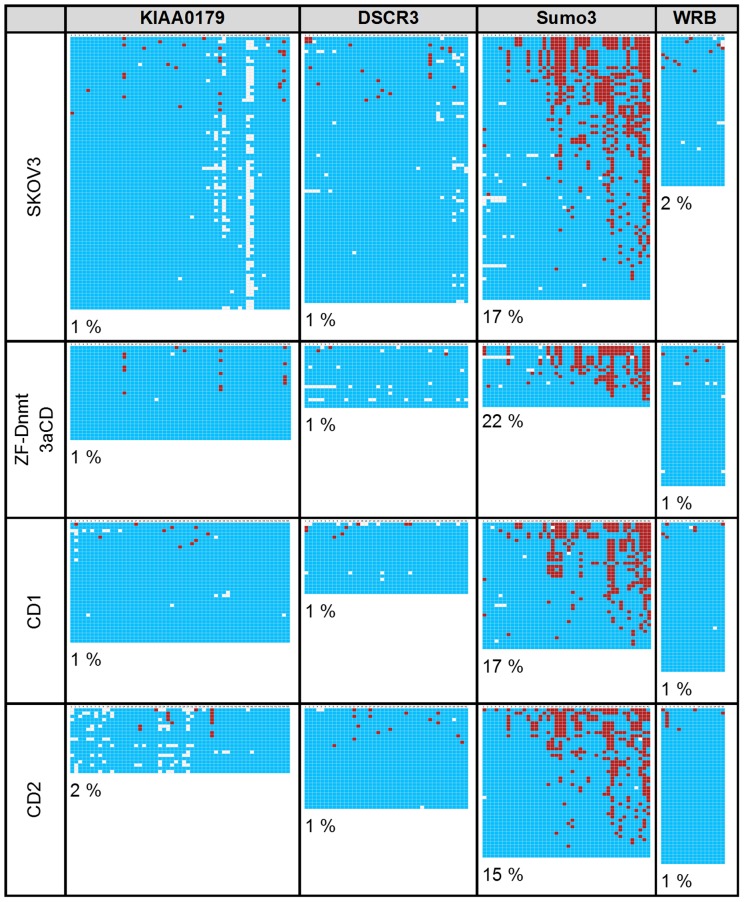
Absence of off-target methylation in SKOV3 cells analyzed by bisulfite sequencing. Methylation of four non-target genes was analyzed (KIAA0179, DSCR3, Sumo3 and WRB). Data presentation is as in Fig. 3. The sequences of the regions analyzed here are given in the Supplementary Information S1.

To analyze methylation at other loci that may accompany our targeted methylation, we investigated the methylation status of four additional non-target genes (KIAA0179, DSCR3, Sumo3 and WRB) as described in the materials and methods. The SKOV3 cells showed a methylation of 1%, 1%, 17%, and 2% respectively, in the four amplicons (Fig. 4 and [62]). SKOV3 cells transiently transfected with ZF-Dnmt3aCD showed methylation levels of 1%, 1%, 22%, and 1% which are almost identical to the results obtained with the untreated SKOV3 cells (Fig. 4). The stable cell lines CD1 and CD2 also showed methylation patterns very similar to the control cells (Fig. 4). While these results do not rule out additional methylation occurring at some individual loci, they show that the methylation of the EpCAM promoter is not accompanied by a massive, unspecific and genome-wide DNA methylation, indicating that targeting was at least partially successful.

### EpCAM expression is repressed by targeted DNA methylation of EpCAM gene promoter

**Figure 5:**
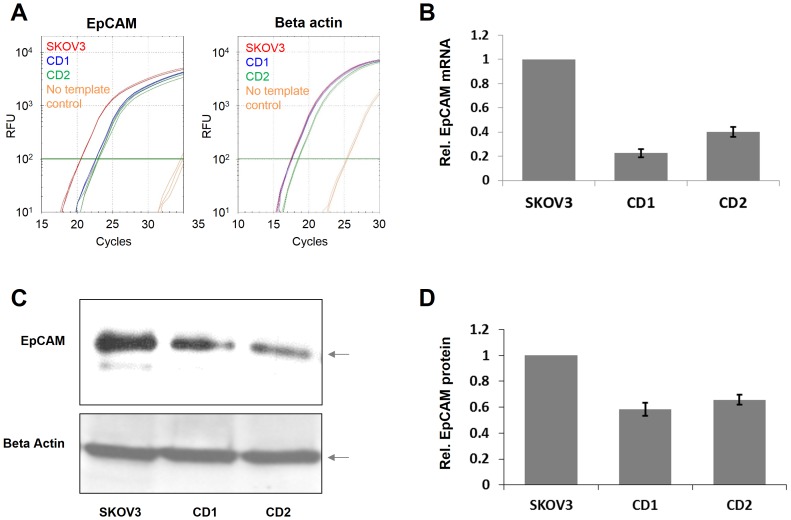
Analysis of EpCAM gene expression after targeted promoter methylation in stable cell lines. A) Example of the RT qPCR analysis of EpCAM (left) and beta actin (right) mRNA amounts in SKOV3 cells and in two independent cell lines which stably express the ZF-Dnmt3a construct (CD1 and CD2). B) Quantification of the RT qPCR analysis of EpCAM expression as shown in panel A. We carried out two independent RNA preparations each analyzed in three technical repeats. The image shows the average of both results, the error bars indicate the standard error. C) Example of the Western blot analysis of EpCAM expression in SKOV3 cells and the CD1 and CD2 stable cell lines (upper panel). Beta actin was used as loading control (lower panel). The EpCAM and beta actin bands are marked with arrows. D) Quantification of the Western Blot analysis of EpCAM expression as shown in panel C. The figure shows an average of two independent experiments, the error bars indicate the standard deviation of the data.

To determine if the promoter methylation led to transcriptional silencing of the EpCAM gene expression, total RNA was isolated from the SKOV3 cells and the stable cell lines CD1 and CD2 and the EpCAM mRNA levels were determined by quantitative RT-PCR. As shown in Fig. 5A and B, we observed a reduction of EpCAM expression of 80% in stable cell line CD1 and 60% in stable cell line CD2. The EpCAM suppression by targeted DNA methylation was confirmed at the protein level by western blotting, where we observed a corresponding reduction of EpCAM expression in the stable cell lines compared to SKOV3 cells (Fig. 5C and D).

### Decreased EpCAM expression associates with a reduction of cell proliferation

**Figure 6:**
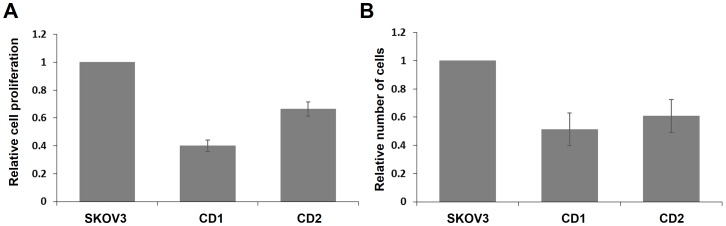
Down regulation of EpCAM expression inhibits the proliferation of SKOV3 cells. A) Results of CCK8 cell proliferation assays conducted with the CD1 and CD2 stable cell lines and SKOV3 cells as reference. The results plotted are from four independent experiments and the error bars indicate the standard error of the mean. B) Viable cell counting performed by Trypan blue staining. The graph represents the data from two independent experiments and the error bars indicate the standard error of the mean.

To investigate whether the reduced EpCAM expression affected the proliferation of SKOV3 cells, an equal number of untreated cells as well as CD1 and CD2 cells were seeded and a cell proliferation assay was performed after three days. For this, the cells were treated with the CCK8 reagent dissolved in cell culture medium and incubated for 4 hours in the incubator. During this time, the reagent can diffuse into the cells, where it is reduced by cellular dehydrogenases to produce an orange colored formazan product, which can be detected by absorption at 450 nm. Since the amount of the formazan is directly proportional to the number of living cells, this assay allows an easy estimate of the number of live cells in the sample. As shown in Fig. 6A, there was 60% reduction of live cells in CD1 and 40% in CD2 when compared to SKOV3 cell control, which is in a very good correlation with the reduction of EpCAM expression in both cell lines. To confirm these results, we also conducted viable cell counting using Trypan blue, as described in materials and methods. For this an equal number of cells were seeded and incubated for four days. Afterwards, the total number of viable cells were counted. As shown in the Fig. 6B, the results corroborated the cell proliferation assay, because there was an about 50% reduction in the number of live cells in CD1 and about 40% in CD2. We conclude that the down regulation of EpCAM leads to a significant reduction of the proliferative potential of SKOV3 cell in vitro.

## Discussion

Targeted DNA methylation by fusion of a DNA methyltransferase domain (here the catalytic domain of Dnmt3a) and a targeting device (here a designed Zinc finger) is an attractive approach for gene silencing [46]–[48]. There are some previous examples of the successful application of this method to achieve gene silencing in human cells [60]–[62]. Here, we demonstrate the targeted methylation and gene silencing of the EpCAM gene, which is a promising target in tumor therapy. We show absence of off-target effects at all loci that were inspected for this, which indicates that targeting was (at least partially) successful. Our results support the notion, that targeted methylation and gene silencing is a universal approach with broad applicability. Furthermore, we demonstrate that the silencing of EpCAM expression leads to a reduction in the proliferative potential of SKOV3 cells.

In a recent paper, der Gun et al. (2013) reported an about twofold silencing of EpCAM after the expression of a Zinc finger fused repressor Kruppel-associated box (SKD) domain, which was delivered with a retroviral vector [38]. Interestingly, this led to a reduction of proliferation in breast cancer cells, but not in SKOV3 cells. Similarly, an RNAi based silencing of EpCAM in SKOV3 cells did not reduce cell proliferation in this work. These results with SKOV3 cells are not in agreement with our data, but there are important differences in the setup of both studies. First of all, van der Gun et al. (2013) used a repression domain, while we employed a DNA methylation mediated epigenetic silencing mechanism. In fact, EpCAM silencing was slightly stronger in our setup, 60–80% in our cell lines vs. 50% observed by van der Gun et al. (2013), which may influence the results. Second, van der Gun et al. (2013) delivered their silencing construct with retroviral vectors and use empty vectors as control. Cell proliferation was assayed 4–6 days after transduction. Our study employed cell lines which express the silencing constructs in a stable manner after an initial transient transfection. Both methods have their advantages and disadvantages. In the retroviral delivery, cellular effects are measured few days after a retroviral infection, which may strongly affect the cellular responses. In addition, in the stable cell line, EpCAM was silenced for several weeks before the cell proliferation analysis was conducted, which gave the cell long time to respond to the reduction of EpCAM expression. In contrast, the proliferation tests of van der Gun et al. (2013) were performed 4 to 6 days after transduction and in the RNAi experiments the readout was done 1 to 3 days after siRNA transfection, which may be another reason for the difference in results. It is one disadvantage of the stable cell line approach that individual cell lines are studied which may have accumulated special adaptations. However, the fact that both stable lines studied here showed similar effects, partially addresses this concern. We conclude that further experiments will be needed to resolve this issue, but the reduction of the proliferative potential of a tumor cell line observed here after epigenetic silencing of the EpCAM promoter is a promising result for potential therapeutic applications of EpCAM silencing in the treatment of cancers with EpCAM overexpression.

For future applications of targeted gene silencing, the efficiency of the delivery of the targeted methyltransferase construct must be improved; several viral delivery strategies are available to this end and are currently developed in our lab and at other places [61], [65]. After the development of several active and functional chimeric methyltransferases that work in cell culture models, it will be one next critical milestone of the future work to integrate these enzymes into an efficient delivery system that allows infection of tumor cells in the animal (or human) body, and leads to inhibition of tumor development in animal models. If this can be achieved, it will also be necessary to determine the potential off-target methylation on a genome wide scale and in a quantitative manner in different tissues. For this, several genome wide DNA methylation analysis methods are available. Depending on the amount of off-target methylation and the affected loci, a risk analysis will allow to assess if these reagents could be safe for clinical trials. If needed the specificity of targeting could be improved by using Zinc fingers modules which recognize longer sequences than used here. Eventually other targeting methods, like TAL effector domains or CRISPR derived methods may be employed, although for them specificity is an issue as well.

## Supporting Information

Information S1 **Primer and amplicon sequences.**
(PDF)
