# Supplementary material for: Targeted Methylation of the Epithelial Cell Adhesion Molecule (EpCAM) Promoter to Silence Its Expression in Ovarian Cancer Cells
Source: PLoS One. 2014 Jan 29;9(1):e87703. doi: 10.1371/journal.pone.0087703 (PMC3906225; doi:10.1371/journal.pone.0087703)
Supplement: Information S1 — Primer and amplicon sequences. (PDF) [file pone.0087703.s001.pdf]

Targeted methylation of the epithelial cell adhesion molecule (EpCAM) promoter to silence its expression in ovarian cancer cells

Suneetha Nunna, Richard Reinhardt, Sergey Ragozin, & Albert Jeltsch

Supplementary information S1: Primer and amplicon sequences

Sequences of primers used for bisulfite DNA methylation analysis

Gene name	Primer sequence
KIAA0179	For: 5’- TTYGTTTTTTTTTGGTTAAGTTT-3’
	Rev: 5’-RCCCACCTATCTCCCTCTA-3’
DSCR3	For: 5’-TTTTTAAGTTTTAGAGTTTGGGGGT-3’
	Rev: 5’ATACCTACCCTCCTCAAATATCCTTAA-3’
SUMO3	For: 5’-GGGTTTTGGGGATTTTTGTT-3’
	Rev: 5’-AACCTCCCAATACCTCAATTTCT-3’
EpCAM	For: 5’-CTTTTTAAGGTTTTAGAGTAG-3’
	Rev: 5’-AAAAAATAAATAAACTCCCCTCCC-3’
WRB	For: 5’-GTAAGTGGTTGTTTGGTTTTTTAA-3’
	Rev: 5’-AAACACTAAAAACTCCCAATATAAAAT-3’

Amplicon sequences

KIAA0179

TAACCCTGGCTAACCTCGCCGTCTACAGCCTGAATTTTGGCAACCGAAAGGCAGCGCC
GGCGCCACGTGCACACGGGCTGGGCCGCTCCGCCAGCTGCCAGGGCCACTGCCGCG
CTCACTCCCAGAGCGCGCTGCGAGCCGCGGCGCCTTTGTGACGCCATCAGCCCGCGC
GCCGCCGCCGCCGCCTTCTGTGCAGTCGCGGCCCGGGCGGACGGTGGCTGGCTGCT
CCGCAGCGCTCGGCTGGCTGCAGCGGCACCGCGGGTTGCGCGGCCGGGGATGCTCC
AGCGGGCGCGATGGCCCCCGCCATGCAGCCGGCCGAGATCCAATTTGCCCAGCGGCT
GGCGTCCAGCGAGAAGGGCATCCGGGACCGAGCGGTGAAGAAGCTGCGCCAGTACAT
CAGCGTGAAGACG

DSCR3

CGTGCCTTTCCACCCTTCACGCGGGGCGCACGGATCTGAATATTTAACACCCGGTTCCA
GGACACGCGCCTGGGGAACACCCACAATCCCTCGCGGCCCGCGGAGCCCGGCTGGC
GGGTGACCCAGCGCCCGCGGCTCCGAGAGGAGCGAGGTCACCCGGCGCTTCCCGAG
ACCTTGGCCCTAGGCCCAGCTGGCGCTGGGACGCGAGGCAACCGAGCCGCCGGCCAA
TCAGCGCTTTGCAGGACTAGCACGCAGGGGCCGCACCCCTGAGGGTGACCGGAACCG

CTCCCGCGGGAAGGGCAGGGTGCGGCGCCTGGCCGCAGACAGACAAGGGGGCGCCG
GCTCTTCAGAACCCCAGGGAGAAAGCGGTGGAGGGCTAGAGTTGAGGAGA

SUMO3

CGCCTCCCCGCGTCGCCCGGGCCCCGACTCCTTTCCTGGGGGCCGCGTTCCTGGGGC
GCCGGGAAGTGAGGCCGGGGCCGCCGCCCATGGCCGCAGGGACCTGGGGCGGAGGA
GGGAGGACGCTGGGGGTGACGTTCCCTCTCAGGAGCCGCGGACAGAGCGGAGAAGG
CGGCCCGCGGGGTCCCGTCCGCGGGGGCCGCGCGAATCGGGCCAGCACCCTAGACG
ATTGGGGGCGCCACCCGCCTCCCTGCCGCCGCGGTTTCCTGTCAGCCCCAGGCCGGG
CTCCTTTGGCCAACGCTCCTACGGGGCTGCCCCGGGCCCTCCACACCCGTCCTCAAGA
AGACCCGTTTTACCTGTGA

WRB

GGGCCGGTGGGGATGCCGCCCCAGTCCCCCGGCGGGTCTGGCGTAGGTACAGGGGT
CTCAACTGGGCGACTGAAGGCCGTAGTAGCGTCTTGGTTGGTCCGTAAGCTTTTTTGAG
ATAGTTGTTAGCGTCTAAAAGGTACGACGCTTTACCCCAAAATCAGACTTTCTGGGTTCT
AGGGGGTTGGGAGAAACACCGGGCAACCCTGGACTCTTCTGGCTGTGGGTGGCGGCG
GCGA

EpCAM

TTCTTCAAGGCTTCAGAGCAGCGCTCCTCCGGTTAAAAGGAAGTCTCAGCACAGAATCT
TCAAACCTCCTCGGAGGCCACCAAAGATCCCTAACGCCGCCATGGAGACGAAGCACCT
GGGGCGGGGCGGAGCGGGGCGCGCGGCCCACACCTGTGGAGAGGGCCGCGCCCC
AACTGCAGCGCCGGGGCTGGGGGAGGGGAGCCTACTCACTCCCCCAACTCCC

(the Zinc finger binding site is colored)
